# Cross-cancer homologous recombination deficiency prediction from whole slide images using transfer learning

**DOI:** 10.1038/s41598-026-52094-6

**Published:** 2026-05-12

**Authors:** Zhengxiao Wang, Ning Jiang, Ruijian Guo, Xiaoming Li, Wei Ye, Shuang Yang

**Affiliations:** 1 School of Life Sciences, Fudan University, 2005 Songhu Road, Yangpu District, Shanghai, China; 2 Amoy Diagnostics Co.Ltd, 39 Dingshan Road, Haicang District, Xiamen, China; 3 Shanghai Xiawei Medical Laboratory Co.Ltd, 138 Xinjun Ring Road, Minhang District, Shanghai, China; 4 Department of Pathology, Anhui Chest Hospital, 1867 Lianhua Road, Hefei, Anhui China

**Keywords:** Homologous recombination deficiency, Deep learning, Transfer learning, Whole slide images, Model interpretable, Cancer, Computational biology and bioinformatics, Oncology

## Abstract

Accurate prediction of Homologous Recombination Deficiency (HRD) is vital for personalized cancer therapy, yet genomic assays are often costly and complex. Predicting HRD from Hematoxylin and Eosin (H&E) stained whole-slide images (WSIs) via deep learning offers an alternative, but model generalization across diverse cancer types remains challenging. We developed a three-stage transfer learning framework to improve HRD prediction across cancer types. After establishing baseline models on data from eight cancer types, the TCGA-BRCA model was selected as a pretrained model for transfer learning applied to eligible cohorts. Transfer learning enhanced HRD prediction performance specifically in cancers with histological similarity to the TCGA-BRCA source model. The AUC increased by + 4% (TCGA-LUAD), + 5% (CPTAC-LUAD), and + 1% (SARC) compared to baseline models. Interpretability analysis confirmed that the model’s predictions were driven by histologically relevant tumor regions, which was further supported by quantitative cellular analysis showing that HRD-high status was characterized by a significantly higher density of neoplastic cells (HRD-high: median 16.0, IQR 1.0–27.0 vs. HRD-low: median 1.0, IQR:0.0–5.0; *p* < 0.001), whereas HRD-low cases featured a stromal-rich microenvironment. **Conclusions** This study demonstrates that transfer learning can enhance HRD prediction from H&E images for cancer types sharing histopathological features with the TCGA-BRCA source model, offering a more efficient and accessible approach for clinical HRD assessment.

**Supplementary Information:**

The online version contains supplementary material available at 10.1038/s41598-026-52094-6.

## Introduction

Impaired DNA damage repair represents one of the hallmarks of cancer, driving cancer evolution^1,2^. Homologous recombination repair (HRR), one of the fundamental DNA repair mechanisms, plays a critical role in maintaining genomic stability by accurately rectifying severe forms of DNA damage such as double-stranded DNA breaks (DSBs) and interstrand cross-links (ICLs)^3–5^. When HR is impaired—due to germline or somatic mutations in HR-related genes (e.g., BRCA1/2), epigenetic modifications, or dysregulation of the HR repair (HRR) pathway—homologous recombination deficiency (HRD) ensues^6–9^. HRD induces selective sensitivity to platinum compounds and poly (ADP-ribose) polymerase inhibitor (PARPi), as evidenced by clinical trials underscoring the critical role of PARPi in enhancing disease-free survival by boosting platinum sensitivity, particularly in ovarian and breast cancers^10–13^. However, the advantages of PARPi therapy are notably limited by the difficulties in diagnosing HRD, as current HRD assessment methods remain controversial regarding their comprehensiveness.

HRD biomarkers can be categorized into genomic, mutation, and functional signatures, each detected through distinct methodologies. Genomic scars which evaluate HRD by analyzing chromosomal aberrations such as Telomeric Allelic Imbalance (TAI)^14^, Loss of Heterozygosity (LOH, > 15 Mb)^15^, and Large-Scale Transitions (LST, breaks between adjacent segments of > 10 Mb)^16^, are assessed via single nucleotide polymorphism (SNP) arrays or next generation sequencing (NGS) methods, including targeted panels like MyChoice CDx (Myriad Genetics Laboratories, Inc., Salt Lake City, UT) and FoundationOne CDx (Foundation Medicine, Inc., Cambridge, MA), shallow WGS^17^, and whole-genome sequencing (HRDetect, CHORD)^18,19^. BRCA/HRR gene mutations (e.g., BRCA1/2, PALB2, RAD51) are identified through targeted sequencing (BRACAnalysis CDx^®^) or comprehensive NGS panels, with FoundationOne CDx also detecting somatic variants^20^. Mutational signatures, particularly single base signature 3 (SBS3), are analyzed using whole genome/exome sequencing-based tools such as MUTation AnaLysIS toolkit (Mutalisk) or Signature Multivariate Analysis (SigMA), which correlate specific patterns with HRD^21,22^. Functional assays, such as RAD51 foci formation, directly measure HR repair capacity but remain experimental due to technical challenges^23^.

In recent years, advancements in artificial intelligence have enabled the extraction of features from whole slide images (WSIs) to predict genetic alterations in digital pathology^24^, such as tumor mutational burden (TMB)^25^, microsatellite instability (MSI)^26^, and gene mutations^26^.In the context of predicting HRD using WSI, previous studies have primarily focused on breast and ovarian cancers^27,28^. However, when applying these models to other cancer types, researchers use models trained on BRCA-related data directly to predict HRD^29^. The strategy overlooks cancer-specific histological characteristics of HRD in various cancer types, resulting in suboptimal performance compared to models trained specifically on the eligible cancer type.

While NGS-based methods remain the current standard for HRD detection, they present several practical limitations that restrict their widespread clinical adoption. These include high costs, lengthy turnaround times, substantial tissue requirements for comprehensive genomic profiling, and limited accessibility in resource-constrained settings. In contrast, WSI-based approaches leverage routinely collected histopathology specimens, offering significant advantages in terms of cost-effectiveness, rapid processing, preservation of tissue for additional testing, and broader availability in standard clinical workflows. Furthermore, digital pathology enables retrospective analysis of archival samples without the need for fresh tissue collection, facilitating large-scale biomarker discovery and validation. These practical benefits position WSI as a promising complementary or alternative platform for HRD assessment, particularly in settings where comprehensive genomic testing is not readily accessible. Beyond histological classification, WSI captures quantitative morphological features at multiple scales—tissue architectural patterns, microenvironmental composition, and cellular-level details such as nuclear morphology and chromatin texture—that directly reflect the genomic instability characteristic of HRD. These subvisual features provide biological information not accessible through qualitative histological assessment alone, enabling richer biomarker prediction from routine H&E slides.

In this study, we present a novel deep learning (DL) framework aimed at improving the prediction of HRD status across various cancer types using HE-stained pathology images. The adoption of transfer learning in our approach addressed the challenges posed by cancer-specific heterogeneity and the scarcity of HRD-labeled data. On the other hand, our framework has the potential to integrate both the shared histological features between cancers and cancer-specific features for HRD prediction, which could improve model performance.

## Results

### Study overview

**Fig. 1:**
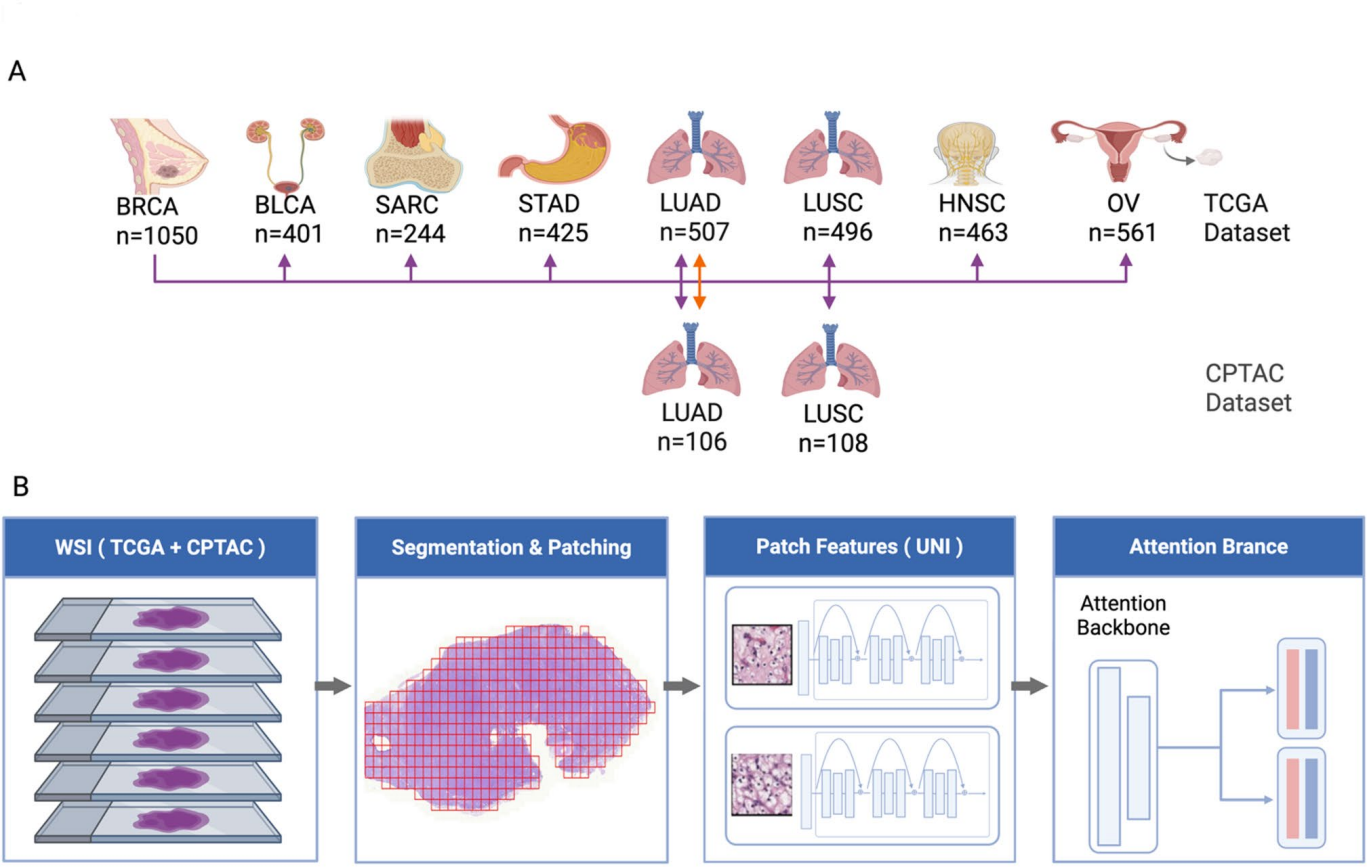
Overview of data cohorts and data analysis workflow. (**A**) Overview of the candidate data cohorts composed of two major cancer databases: The Cancer Genome Atlas (TCGA) encompassing 8 distinct cancer types (*n* = 4,147 patients) and the Clinical Proteomic Tumor Analysis Consortium (CPTAC) containing 2 carcinoma subtypes (*n* = 214 patients). Created in BioRender.com. (**B**) The CLAM-SB model and analysis workflow used in our study. **Abbreviations**: BRCA, breast invasive carcinoma; BLCA, bladder urothelial carcinoma; SARC, sarcoma; STAD, stomach adenocarcinoma; LUAD, lung adenocarcinoma; LUSC, lung squamous cell carcinoma; UCEC, uterine corpus endometrial carcinoma; HNSC, head and neck squamous cell carcinoma; OV, ovarian serous cystadenocarcinoma.

**Fig. 2:**
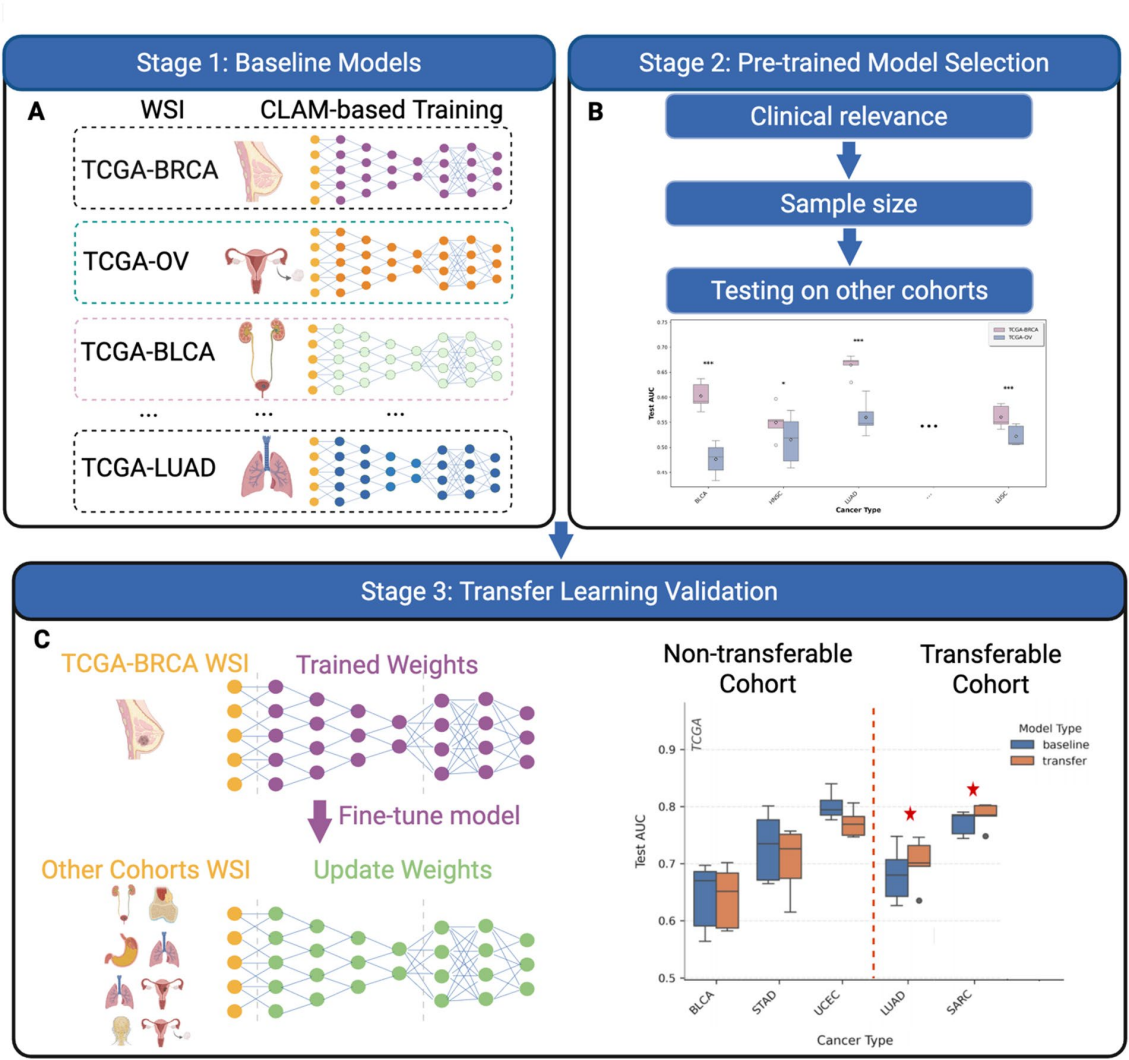
Illustration of three-phase framework. **(A)** Baseline model training for candidate cancer types: UNI-pretrained feature extractor with randomly initialized CLAM-SB MIL network trained from scratch. **(B)** Pretrained models screening for transfer learning. **(C)** Comparative performance analysis between baseline models and fine-tuned TCGA-BRCA models across cancer types. Red stars denote the eligible cancers in the screening phase. **Abbreviations**: BRCA, breast invasive carcinoma; BLCA, bladder urothelial carcinoma; LUAD, lung adenocarcinoma; STAD, stomach adenocarcinoma; UCEC, uterine corpus endometrial carcinoma; SARC, sarcoma; TCGA, The Cancer Genome Atlas; CPTAC, Clinical Proteomic Tumor Analysis Consortium.

We developed a three-stage transfer learning framework for cross-cancer HRD prediction from H&E whole-slide images (Figs. 1 and 2). Fig. 1 provides an overview of the data cohorts spanning eight cancer types from TCGA and CPTAC (4,361 patients, 11,084 WSIs), and Fig. 2 illustrates the three-phase analytical workflow encompassing baseline model establishment, source model selection, and transfer learning implementation. The HRD score was calculated using scarHRD, an R package that quantifies genomic scars through three DNA-based hallmarks: loss of heterozygosity (LOH), large-scale state transitions (LST), and telomeric allelic imbalance (NtAI). A validated cutoff score of ≥ 42 was applied to classify samples as HRD-high or HRD-low, as detailed in Section  “HRD label” of the Methods. Applying this common HRD score cutoff across cancer types resulted in variable HRD-high prevalence, ranging from 11.2% in HNSC to 55.8% in OV (Supplementary Fig. 1).

### Baseline model performance: hrd prediction across candidate cancer types

**Fig. 3:**
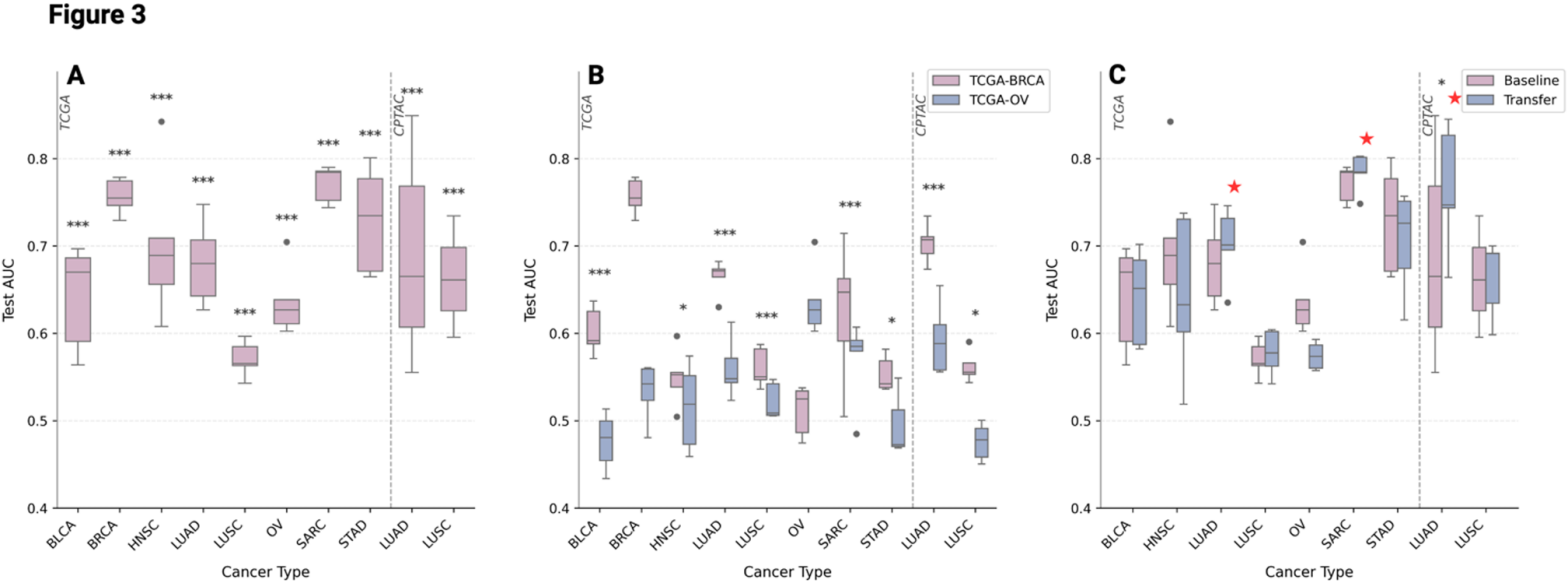
The model performance for HRD status prediction in our study. The area under the receiver operating characteristic curve (AUROC) was used as the main evaluation metric. **(A)** Baseline model performance: cancer-specific models trained from scratch on each candidate cancer type (BLCA, BRCA, HNSC, LUAD, LUSC, OV, SARC, STAD). **(B)** Direct cross-cancer inference: TCGA-BRCA model (pink) and TCGA-OV model (blue), each trained only on their respective cancer type, applied to other cancers without fine-tuning. The vertical dashed line separates TCGA and CPTAC cohorts. **(C)** Transfer learning efficacy: comparison of cancer-specific baseline (pink) and fine-tuned TCGA-BRCA model (blue) performance across cancer types. Red stars indicate significant improvement with transfer learning (*p < 0.05, **p < 0.01, ***p < 0.001). **Abbreviations**: BRCA, breast invasive carcinoma; BLCA, bladder urothelial carcinoma; HNSC, head and neck squamous cell carcinoma; LUAD, lung adenocarcinoma; LUSC, lung squamous cell carcinoma; OV, ovarian serous cystadenocarcinoma; SARC, sarcoma; STAD, stomach adenocarcinoma; UCEC, uterine corpus endometrial carcinoma.

To evaluate the feasibility of CLAM (Clustering-constrained Attention Multiple Instance Learning)-based prediction of HRD status from histopathology images, the baseline model performance across the TCGA and CPTAC datasets was assessed and summarized as follows. In TCGA cohorts, the model achieved mean AUC ≥ 0.60 in 7/8 cancer types  (Fig. 3A). Three cancers surpassed AUC 0.70: TCGA-SARC (AUC = 0.77, 95% CI: 0.74–0.80, *p* < 0.01), TCGA-BRCA (AUC = 0.75, 95% CI: 0.73–0.78, *p* < 0.01), and TCGA-STAD (AUC = 0.71, 95% CI: 0.68–0.75, *p* < 0.01). Notably, TCGA-LUSC showed the lowest performance (AUC = 0.57, 95% CI: 0.54–0.60, *p* < 0.01). In CPTAC cohorts, the baseline models achieved AUC of 0.69, 95% CI: 0.65–0.75 (*p* < 0.01) for CPTAC-LUAD and 0.66, 95% CI: 0.60–0.71 (*p* < 0.01) for CPTAC-LUSC. While AUROC served as the primary evaluation metric, comprehensive performance metrics including precision-recall curves (PRC), precision, recall, and F1 score are provided in Supplementary Data 3 (Sheet 1).

### Pretrained models selection for transfer learning

To determine the optimal pretrained model for downstream transfer learning, we evaluated the predictive performance of models pretrained on TCGA-BRCA and TCGA-OV across eight independent cancer cohorts.

The model trained on the TCGA-BRCA dataset generally exhibited more robust and consistent cross-cancer generalizability. It achieved AUC values exceeding 0.60 in several cancer types, including LUAD (AUC = 0.65, 95% CI: 0.63–0.66), SARC (AUC = 0.62, 95% CI: 0.60–0.63), and CPTAC-LUAD (AUC = 0.68, 95% CI: 0.66–0.71) (Fig. 3B). In contrast, the model based on the TCGA-OV dataset showed comparatively lower discriminatory ability in most external tests, with AUCs below 0.60 for multiple cancer types, such as BLCA (AUC = 0.48, 95% CI: 0.45–0.50) and STAD (AUC = 0.49, 95% CI: 0.48–0.51) (see also Fig. 3B). Statistical significance was consistently observed for the BRCA-based model across several cancer types, such as LUAD (*p* = 1.27e-53) and SARC (*p* = 1.76e-33), whereas the predictions from the OV-based model did not reach statistical significance (*p* > 0.05) in cancers like HNSC (*p* = 0.75) and STAD (*p* = 0.27). These findings indicated that the TCGA-BRCA pretrained model offers superior and more reliable generalizability, supporting its selection as the source model for subsequent transfer learning tasks.

### Model performance using transfer learning

To evaluate transfer learning efficacy in biologically aligned cohorts, we compared model performance across candidate cancer types between approaches with (i.e., fine-tuned TCGA-BRCA models) and without (i.e., baseline models) BRCA model transfer (Fig. 3C). In LUAD, fine-tuned TCGA-BRCA models significantly improved performance for both TCGA-LUAD (transfer learning: AUC = 0.69, 95% CI: 0.67–0.70; baseline: AUC = 0.65, 95% CI: 0.61–0.68) and CPTAC-LUAD (transfer learning: AUC = 0.74, 95% CI: 0.72–0.76; baseline: AUC = 0.69, 95% CI: 0.65–0.75), suggesting conserved HRD-shared features between BRCA and LUAD. Similarly, TCGA-SARC showed marginal gains with transfer learning (AUC = 0.78, 95% CI: 0.77–0.80 vs. baseline AUC = 0.77, 95% CI: 0.74–0.80). For other cancer types (TCGA-BLCA, TCGA-HNSC, TCGA-LUSC, CPTAC-LUSC, TCGA-OV, TCGA-STAD), baseline models outperformed fine-tuned counterparts (*p* < 0.05), indicating that transfer learning could enhance performance only in biologically or morphologically HRD-related cancers (e.g., BRCA-LUAD/SARC).

### Cross-dataset validation transfer learning performance

To assess the generalizability of the transfer learning framework across independent datasets, we conducted bidirectional cross-dataset validation experiments (Supplementary Fig. 2 and Supplementary Data 3). We trained baseline models (trained from scratch on the target dataset) and transfer learning models (pretrained on TCGA-BRCA and fine-tuned on the target dataset) under four scenarios: (i) TCGA-LUAD→CPTAC-LUAD, (ii) TCGA-LUSC→CPTAC-LUSC, (iii) CPTAC-LUAD→TCGA-LUAD, and (iv) CPTAC-LUSC→TCGA-LUSC.

Transfer learning performance varied depending on the transfer direction and cancer subtype (Supplementary Fig. 2; detailed data in Supplementary Data 3, Sheet 2). In the TCGA→CPTAC direction, baseline models outperformed transfer learning models for both LUAD (baseline AUC = 0.72, 95% CI: 0.66–0.79] vs. transfer AUC = 0.69, 95% CI: 0.62–0.77) and LUSC (baseline AUC = 0.60, 95% CI: 0.53–0.66 vs. transfer AUC = 0.56, 95% CI: 0.53–0.59).

Conversely, in the CPTAC→TCGA direction, transfer learning models achieved superior performance for LUAD (transfer AUC = 0.69, 95% CI: 0.67–0.70 vs. baseline AUC = 0.63, 95% CI: 0.61–0.66), while showing comparable performance for LUSC (transfer AUC = 0.55, 95% CI: 0.53–0.56 vs. baseline AUC = 0.53, 95% CI: 0.50–0.56). Notably, the CPTAC→TCGA (LUAD) scenario represented the only cross-dataset setting where transfer learning conferred a clear advantage, with a 6-percentage-point improvement in mean AUC.

### Comparison with prior multi-cancer approach

To validate that our transfer learning strategy offers advantages over conventional multi-cancer modeling, we performed a head-to-head comparison with a prior approach^29^, which trained independent models on each cancer type without cross-cancer knowledge transfer. Using identical data splits and their publicly available code [https://github.com/KatherLab/marugoto.git], we compared three strategies: (i) the prior independent training, (ii) our independent training, and (iii) our BRCA pre-training with cancer-specific fine-tuning. The re-implementation results and per-cancer comparisons are presented in Supplementary Data 4 (Sheets 1 and 2), with group-level summaries by transfer-responsiveness in Sheet 3.

The per-cancer results (Supplementary Data 4, Sheet 2) show that our transfer learning achieved comparable or superior performance in specific contexts. In LUAD, our transfer learning (TCGA: AUC = 0.69, 95% CI: 0.67–0.70; CPTAC: AUC = 0.74, 95% CI: 0.72–0.76) matched or exceeded the prior independent training (TCGA: AUC = 0.69, 95% CI: 0.63–0.74; CPTAC: AUC = 0.71, 95% CI: 0.55–0.86), while achieving superior cross-dataset generalization. In SARC, our transfer learning (AUC = 0.78, 95% CI: 0.77–0.80) outperformed the prior approach (AUC = 0.71, 95% CI: 0.62–0.79) by 0.07. For other cancer types, transfer learning did not improve performance over independent training, with our baseline and the prior approach generally achieving higher or equivalent AUCs.

The results by transfer-responsiveness group (Supplementary Data 4, Sheet 3) reveal that the transfer-responsive group (LUAD, SARC, CPTAC-LUAD) achieved a mean AUC of 0.74 with our transfer learning, compared to 0.70 for the prior approach and 0.71 for our baseline; the non-transfer-responsive group (BLCA, HNSC, LUSC, OV, STAD, CPTAC-LUSC) showed comparable performance across strategies (0.62–0.65). Collectively, these results confirm that BRCA pre-training with cancer-specific fine-tuning represents a viable alternative to conventional single-cancer training in biologically aligned contexts, consistent with our internal findings (Fig. 3C).

### Validation of HRD predictions from genomics and histopathology

**Fig. 4:**
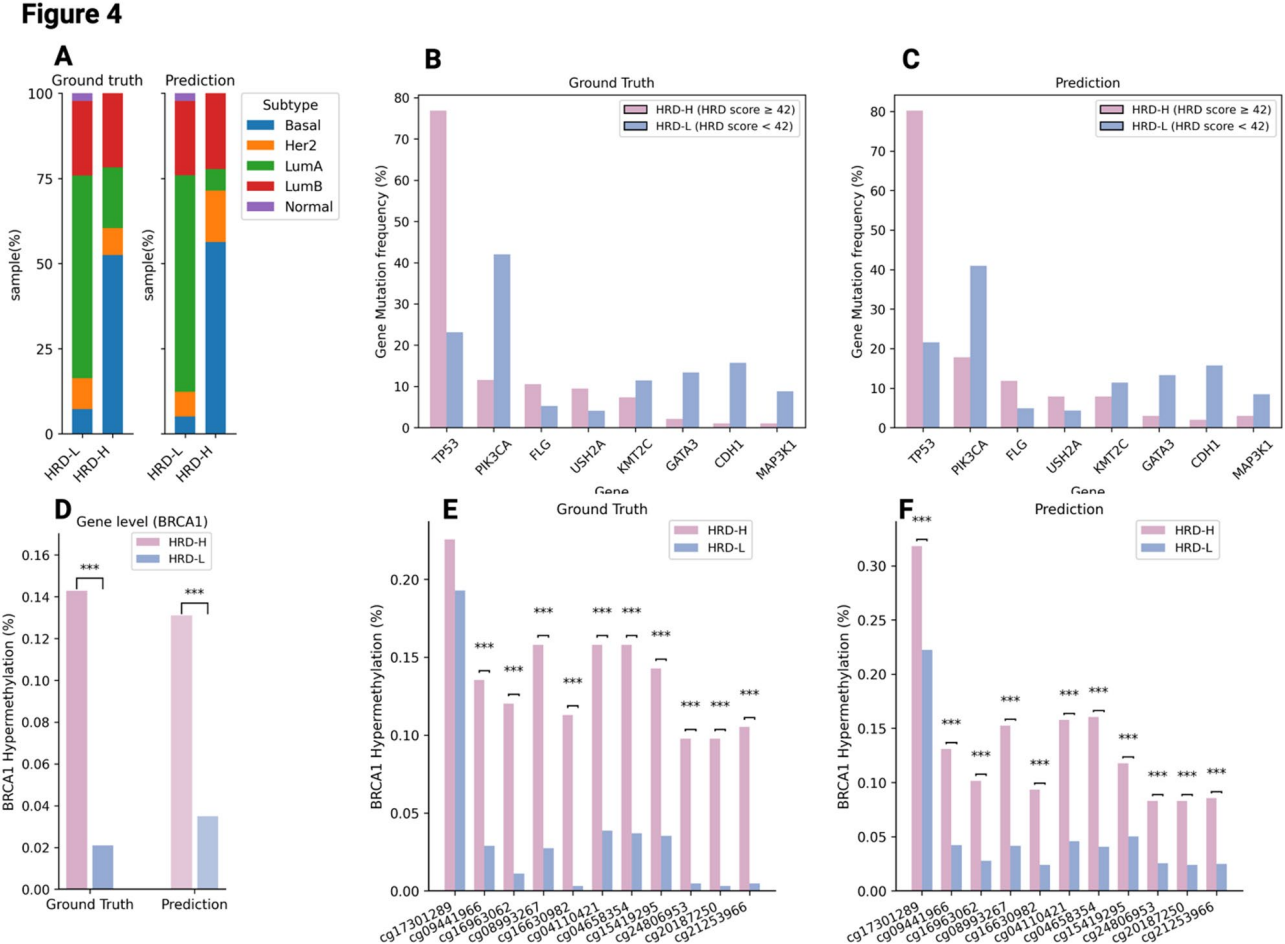
Molecular Characterization of The Cancer Genome Atlas breast cancer (TCGA-BRCA) cohorts. Ground truth HRD labels were determined using a threshold of HRD score ≥ 42 for HRD-H and HRD score < 42 for HRD-L across all cancer types. **(A)** Distribution of PAM50 molecular subtypes (Basal, Her2, LumA, LumB, Normal) within HRD-H and HRD-L groups for both ground truth labels (left) and model predictions (right). (**B**, **C**) Gene mutation frequencies stratified by ground truth (**B**) and predicted (**C**) HRD status. (**D–F**) BRCA1 promoter methylation patterns: (**D**) gene-level comparison between ground truth (left) and predicted (right) HRD stratification; (**E**, **F**) probe-level methylation for (**E**) ground truth and (**F**) predicted HRD status. Methylation levels of individual CpG probes (e.g., cg09441966) within the BRCA1 promoter region are shown, with beta values indicating methylation intensity. P-values calculated by chi-square test (**D**) or Wilcoxon rank-sum test (**E**–**F**); *p < 0.05, **p < 0.01, ***p < 0.001.

Our analysis began by confirming the biological validity of the HRD-H and HRD-L subgroups defined by our model. We first examined the distribution of PAM50 molecular subtypes within HRD-H and HRD-L groups for both ground truth labels and model predictions (Fig. 4A). While Basal-like subtype was enriched in both ground truth and predicted HRD-H groups, a substantial proportion of HRD-H cases belonged to other subtypes—specifically, 52% of ground truth HRD-H cases and 55% of predicted HRD-H cases were non-Basal (Her2, LumB, LumA, or Normal). Conversely, a notable fraction of Basal-like tumors were classified as HRD-L. This indicates that HRD status and molecular subtype are not synonymous, supporting the model’s ability to capture HRD-specific features independent of subtype classification. Supplementary Fig. 3 confirms distinct HRD-associated mutational profiles within each subtype category, indicating the model captures HRD-specific rather than subtype-specific genomic features. PAM50 subtype annotations were obtained from UCSC Xena (PAM50Call_RNAseq).

Analysis of the mutational landscapes in the TCGA-BRCA cohort revealed distinct genomic profiles between these two subgroups for both ground-truth and predicted labels (Fig. 4B, C). In the ground-truth HRD-H group, TP53 alterations dominated (76.84%), statistically significant exceeding frequencies in HRD-L cancers (23.15%, *p* < 0.001), followed by PIK3CA (11.58% in HRD-H vs. 42.00% in HRD-L), FLG (10.53% vs. 5.25%), and USH2A (9.47% vs. 4.06%). Conversely, HRD-L cancers exhibited enrichment of PIK3CA (42.00%), GATA3 (13.37%), CDH1 (15.75%), and MAP3K1 (8.83%), which were rare in HRD-H (≤ 2.11% for all). In the predicted HRD-H subgroup, the model accurately mirrored these trends: TP53 remained the most frequently altered gene (80.20%, vs. 76.84% in ground truth), with low frequencies of HRD-L-associated genes (PIK3CA: 17.82% predicted vs. 11.58% ground truth; GATA3: 2.97% vs. 2.11%). Predicted HRD-L cancers similarly recapitulated ground-truth patterns, showing elevated PIK3CA (40.92% predicted vs. 42.00% ground truth), GATA3 (13.32% vs. 13.37%), and CDH1 (15.74% vs. 15.75%).

To validate that model predictions capture biologically meaningful HRD correlates, we compared BRCA1 promoter methylation patterns between ground truth and predicted HRD groups. As shown in Fig. 4D–F, predicted HRD-H samples exhibited methylation patterns comparable to ground truth HRD-H samples at both gene-level and individual CpG probe-level, confirming the biological validity of model stratification. While Figs. 4 focus on TCGA-BRCA due to its established HRD biology and data completeness, Supplementary Fig. 4 presents validation on TCGA-LUAD using the transfer learning model, demonstrating cancer-type-specific molecular correlates.

**Fig. 5:**
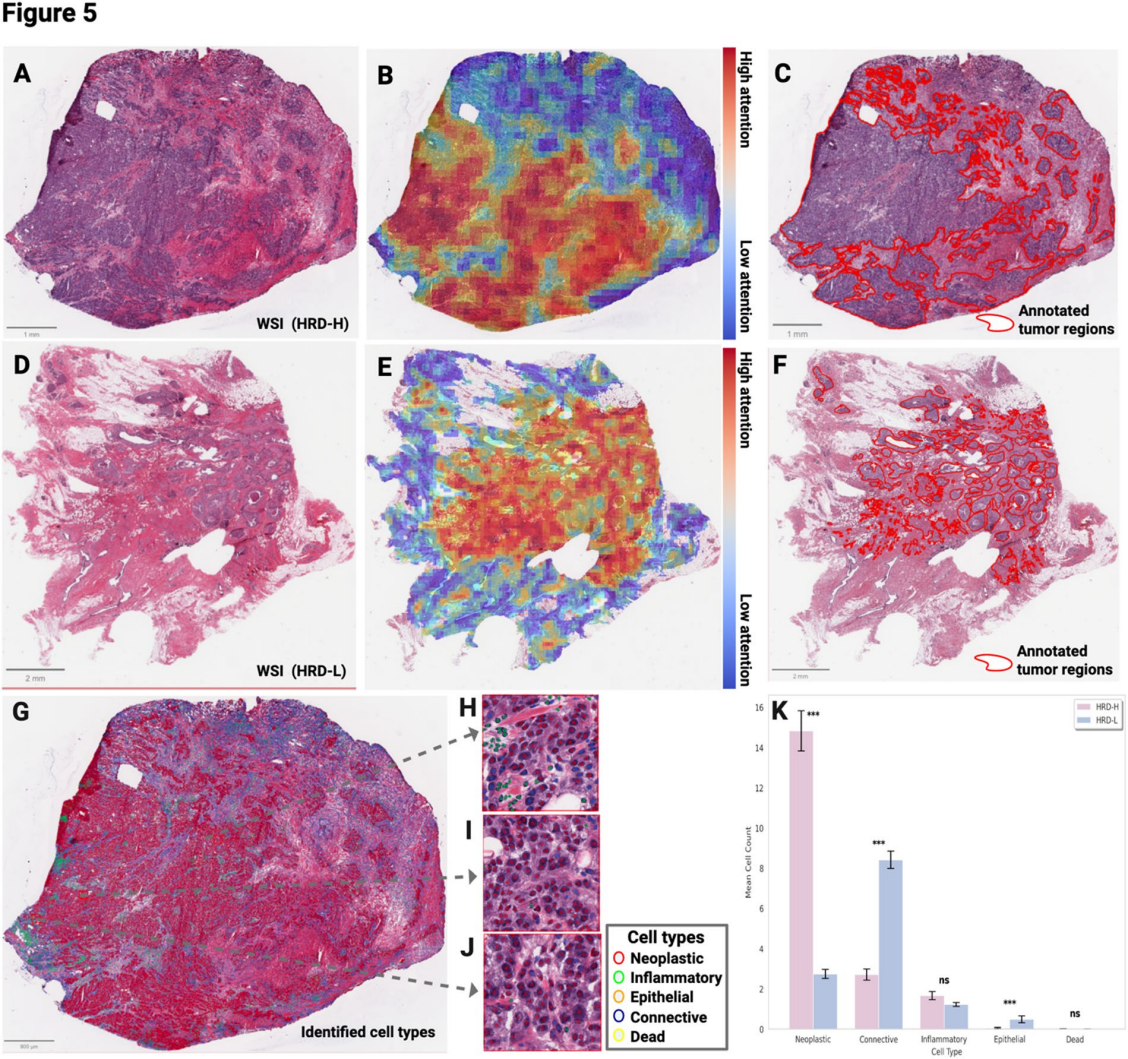
Visualization of model interpretation and cellular analysis of HRD samples. (**A**, **D**) Whole-slide images (WSI) of representative HRD-H and HRD-L samples, respectively. (**B**,** E**) Model-generated attention heatmaps for (A) and (D), with red/blue indicating high/low attention. (**C**, **F**) Pathologist annotations of tumor areas (red outlines) for (A) and (D). (**G**) Cellvit++ analysis of (A), identifying cell types: Neoplastic (red), Inflammatory (green), Epithelial (orange), Connective (dark blue), Dead (bright yellow). (**H–J**) The top three attention tiles from (G) with cell type details. (**K**)Box plots comparing cell type abundances in HRD-H vs. HRD-L groups, analyzed from top-scoring tiles of top-ranked WSIs.

To understand how the model distinguishes these states from histology, we leveraged the inherent attention mechanism of the CLAM-SB framework to generate heatmaps overlayed on the original H&E-stained WSIs. For objective evaluation, the tumor regions of interest (ROIs) were first annotated by an experienced pathologist. We then visually assessed whether the model-attended areas aligned with these annotated regions. A representative example comparing an HRD-H and an HRD-L slide is presented in Fig. 5A and D. Visual assessment of these heatmaps revealed that the model’s focus coincided well with pathologist-annotated tumor regions. Specifically, areas with high attention scores (shown in red on the heatmaps in Fig. 5C and D) were predominantly concentrated within the tumor regions that had been annotated by a pathologist (red outlines in Fig. 5E and F). Conversely, regions corresponding to normal or non-tumor tissue consistently received low attention scores (shown in blue on the heatmaps). This concordance indicates that the model’s decision-making process is correctly focused on histologically relevant tumor regions, rather than being driven by non-specific or background tissue structures.

To further decipher the specific cellular features that underpin the model’s predictions, we performed a quantitative analysis of the tumor microenvironment in the most influential image regions (Fig. 5G-J), enabling a detailed comparison of cellular composition between the groups. The results are summarized graphically in Fig. 5K, with the corresponding tile-level cell counts for each WSI provided in Supplementary Data 1 (Sheet 1). Given the non-normal distribution of cell counts, data are presented as median [Q1, Q3]. Significant differences (*p* < 0.001) were observed in the prevalence of four cell types between HRD-H and HRD-L groups. The most prominent finding was a significantly higher abundance of neoplastic cells in tiles critical for predicting HRD-H status (16.0 [1.0–27.0]) compared to HRD-L (1.0 [0.0–5.0]). Conversely, tiles from HRD-L slides were characterized by significantly higher counts of connective tissue cells (8.0 [4.0–16.0] vs. 1.0 [0.0–4.0]). Notably, both HRD-H and HRD-L high-attention tiles were derived from regions with maximal model attention, yet they exhibited distinct cellular compositions. This indicates that the model identifies HRD status-specific morphological patterns—characterized by high tumor cellularity in HRD-H and stromal enrichment in HRD-L—rather than simply detecting neoplastic regions. In contrast, the counts of inflammatory cells and dead cells showed no significant difference between the two groups (*p* = 0.054 and *p* = 0.157, respectively).

## Discussion

HRD is a crucial biomarker for targeted therapies in solid cancers, such as ovary and breast cancer, significantly impacting treatment outcomes and patient prognosis^20,30^. However, its clinical utility is restricted by current detection approaches, which primarily rely on genomic sequencing^31^. These approaches are costly and less accessible, particularly in resource-limited settings^32–34^. To address these challenges, alternative methods, such as using deep learning models based on H&E slides to identify HRD^28^.

In this study, we applied DL to histological WSIs and show that it can predict HRD status across eight tumor types in the TCGA and CPTAC cohorts. The selection of TCGA-BRCA as the source model for transfer learning was informed by its more robust cross-cancer generalizability compared to TCGA-OV (Fig. 3B). Head-to-head comparison with prior study validated that the performance gains of our framework are specifically attributable to cancer-specific fine-tuning (Supplementary Data 4). While our transfer learning framework—pretrained on TCGA-BRCA and building upon MIL models’ established capability to predict HRD from H&E-WSIs —was successfully applied, its performance varied across tumor types, with notable improvements in certain adenocarcinomas such as LUAD but limited gains or slight declines in histologically distinct cancers like LUSC. Specifically, we recorded AUC improvements of + 4% in TCGA-LUAD, + 5% in CPTAC-LUAD, and + 1% in TCGA-SARC, in contrast to cohorts like TCGA-LUSC, which showed negligible gains or a slight decrease. One plausible explanation for this phenomenon may be the occurrence of negative transfer^35,36^, where knowledge from a less-related source domain interferes with learning in the target domain.

The variable efficacy of transfer learning across cancer types suggests that HRD-related morphology may be partially lineage-specific. The likelihood of negative transfer is largely determined by the histopathological divergence between the source and target domains. This principle explains the observed performance pattern. The gains in LUAD, an adenocarcinoma, can be attributed to its morphological congruence with the BRCA source domain, as both cancer types originate from glandular cells^37^ and share morphological traits with BRCA. Conversely, the stagnation in cohorts like LUSC likely results from its distinct histopathology as a squamous cell carcinoma^38^, which diverges significantly from the glandular features of BRCA, thereby inducing negative transfer^29^. However, our study did not systematically investigate the biological determinants of successful transfer. We speculate that histological similarity may facilitate transfer, but definitive conclusions require future studies with explicit morphological quantification and controlled source-target pairing experiments.

Our molecular analyses further revealed that TP53 alterations are strongly associated with HRD-H cases, while PIK3CA alterations are more frequently observed in HRD-L cases. The association of TP53 mutations with HRD-H aligns with established literature^29,39,40^, which showed TP53 was the most enriched alteration in HRD-H ground truth (67%), contrasting with PIK3CA enrichment in HRD-L (39%). This consistency underscores the biological relevance of our model’s output.

To elucidate the underlying biological basis of the TCGA-BRCA model, we conducted a multi-level interpretability analysis. At the WSI level, attention heatmaps were generated in a manner consistent with previous studies^41,42^. These heatmaps revealed that the model’s high-attention regions corresponded strongly to pathologist-annotated the tumor regions of interest (ROIs)^43^, confirming that these cellular differences are indeed being gleaned from morphologically relevant tissue regions.

Beyond the qualitative WSI-level attention analysis, we performed automated cell segmentation and classification on the high-attention regions to enable a quantitative comparison of the cell composition. This cell-level quantification revealed distinct cellular patterns: In HRD-H cases, high-attention regions exhibited a significantly higher density of neoplastic cells and a concomitant decrease in connective tissue cells, indicating the model’s potential use of high tumor cell density as a primary discriminative feature^28,29^. In contrast, HRD-L status was associated with a stromal-rich microenvironment, characterized by a significantly higher proportion of connective tissue and epithelial cells within the model’s high-attention regions. By establishing a quantitative link between a deep learning model’s attention and specific, measurable cellular features, our study provides novel, cell-specific evidence for the model’s predictions. While our current analysis focused on high-attention tiles, we acknowledge that the relationship between focal high-density regions and global tumor cellularity remains to be fully elucidated. It would be of interest to explore whether whole-slide neoplastic cell density correlates with HRD status and how this relates to the model’s attention-based identification of focal high-density regions. Nevertheless, cell-type proportion differences in high-attention tiles are statistically significant and consistent with HRD biology. The modest absolute values—partly reflecting the small spatial scale of individual tiles (256 × 256 pixels at 20× magnification) and the skewed distribution of cell counts—indicate that the model integrates multiple subtle morphological cues rather than relying on single dominant features. This advance beyond qualitative assessment offers crucial insights into the cellular basis of HRD and strongly validates the biological relevance of our model.

Our study demonstrates the feasibility of predicting HRD across multiple cancer types using transfer learning, but several limitations should be considered. First, to firmly establish the efficacy of our transfer learning approach, models were trained and evaluated within individual datasets (TCGA and CPTAC) rather than in a direct cross-dataset manner. While this controlled design reduces confounding factors, it prioritizes proof-of-concept over an assessment of broad generalizability, which requires future validation with larger, multi-source datasets. Secondly, using the scarHRD score with a threshold of 42 as the genomic ground truth may introduce bias^44,45^, as it can capture non-HRD-related genomic instability. Finally, the interpretability analysis was restricted to a predefined set of cellular phenotypes^46^; future studies incorporating more granular cell states or spatial context could provide deeper insights.

The clinical relevance of our model lies in complementing genomic HRD testing rather than replacing it. By demonstrating that predicted HRD-H recapitulates the mutation landscape and BRCA1 methylation patterns of genomically-defined HRD-H, we establish that morphological features encode functional HRD status. This enables HRD phenotyping in archival tissue where genomic data may be absent or degraded. Although PARPi therapy is currently approved only for ovarian, breast, prostate, and pancreatic cancers, clinical trials are underway for other solid tumors, with additional approvals anticipated. It is well-established that the population of patients with HRD-H significantly outnumbers those with BRCA1/2 mutations across cancer types^19,47^. This underscores the need for complementary biomarkers beyond BRCA testing to identify patients eligible for PARPi. AI-based assays, leveraging easily accessible histology slides, low computational cost, and rapid turnaround, offer a promising solution^48^. They could expand the ability to identify patients who may benefit from PARPi, potentially informing clinical trial design^29^. Furthermore, by aiding in patient screening and enrollment, such methods could accelerate the pace of clinical trials.

## Materials and methods

### Cohort selection and data sources

#### Whole slide images

To establish a robust HRD prediction framework based on H&E slides, we leveraged a large-scale dataset from two publicly available oncology repositories: The Cancer Genome Atlas (TCGA; https://portal.gdc.cancer.gov) and the Clinical Proteomic Tumor Analysis Consortium (CPTAC)^49^. Our study cohort included 4,361 patients, from which a total of 11,084 whole-slide images (WSIs) were analyzed, as multiple slides were available for a subset of patients.

Specifically, to select candidate cancer types from TCGA cohort based on statistical feasibility for downstream modeling, we applied two main criterions: (1) A minimum HRD positivity rate of 10% was set to ensure an adequate number of positive cases and to address potential class imbalance during model training (Supplementary Table 1) and (2) a minimum of ≥ 200 cases per cancer type to maintain adequate sample size for robust model performance. (Supplementary Table 2). We identified 4,147 eligible cases from TCGA, covering eight malignancies (Fig. 1A). These comprised bladder urothelial carcinoma (BLCA, *n* = 401), breast invasive carcinoma (BRCA, *n* = 1,050), sarcoma (SARC, *n* = 244), stomach adenocarcinoma (STAD, *n* = 425), lung adenocarcinoma (LUAD, *n* = 507), head and neck squamous cell carcinoma (HNSC, *n* = 463), ovarian serous cystadenocarcinoma (OV, *n* = 561), and lung squamous cell carcinoma (LUSC, *n* = 496). Next, to validate our transfer learning approach across diverse data sources, we incorporated two overlapping cancer cohorts from CPTAC (*n* = 214; Fig. 1A), including LUAD(*n* = 106), and LUSC (*n* = 108). These cohorts were selected as independent datasets to assess the framework’s suitability, complementing the TCGA cohorts used for initial training.

#### HRD label

To obtain HRD labels, we acquired HRD scores for TCGA and CPTAC cohorts of various cancer types from previous studies^1,29,50^ (Supplementary Data 2). The HRD score was calculated using scarHRD (https://github.com/sztup/scarHRD), an R package that quantifies genomic instability as the unweighted sum of three structural variants^51^: loss of heterozygosity (LOH, > 15 Mb)^15^, large-scale state transitions (LST, breaks between adjacent segments of > 10 Mb)^16^,and number of telomeric allelic imbalance (NtAI)^14^. A validated cutoff score of 42 was used to determine HRD-high (HRD-H, ≥ 42) from HRD-low (HRD-L) status^9^. This threshold was concordance with criteria from pivotal clinical trials^52^ and other studies^53,54^, with strict adherence throughout our analytical pipeline. The distribution of HRD scores and the resulting label proportions for each cancer type are presented in Supplementary Fig. 1.

### WSI preprocessing and dataset splitting

The TCGA dataset comprised both formalin-fixed paraffin-embedded (FFPE) and frozen section specimens, scanned at either 20x or 40x magnifications. In contrast, CPTAC cohorts exclusively utilized frozen tissue sections uniformly digitized at 20× magnification.

To enhance computational efficiency, we first implemented Otsu’s algorithm^55^ to segment tissue from background without requiring prior training data or manual parameter optimization. Secondly, the extracted tissue regions were partitioned into non-overlapping 256 × 256-pixel image tiles at the baseline magnification (20×).Thirdly, we employed a Clustering-constrained Attention Multiple-Instance Learning (CLAM-SB)^56^ framework integrated with the UNI^57^ foundation model—a pan-cancer histopathology encoder pretrained on 100 million histopathology images from 100,000 diagnostic H&E-stained WSIs (77 TB) spanning 20 major tissue types—to perform attention-based feature aggregation. The UNI model was selected because it was pretrained on non-TCGA data, thereby ensuring that there was no potential data leakage when evaluating downstream models on TCGA WSI data. Ultimately, for each patient, tile-wise 1024-dimensional feature vectors were generated across all tissue regions (Fig. 1B). These patient-specific tile embeddings were subsequently aggregated through attention-based pooling to derive unified WSI-level representations, thereby preserving granular morphological patterns while controlling computational complexity.

To ensure methodological robustness, statistical reliability, and comparability of results, a repeated cross-dataset validation framework was employed with strict patient-level splits to prevent data leakage. For each tumor-specific cohort, the dataset was partitioned into five distinct, non-overlapping splits at the patient level. For patients with multiple slides (as detailed in Supplementary Table 2), all tiles from all slides belonging to the same patient were exclusively assigned to either the training, validation, or testing set—never to multiple sets. Within each split, a stratified sampling procedure was applied to allocate cases to training (60%), validation (20%), and test (20%) subsets, preserving the distribution of HRD status across all subsets. This methodology of utilizing five fixed, pre-defined partitions mitigates sampling bias and enhances the statistical robustness of performance estimates while maintaining strict separation between data subsets throughout all experimental phases.

### Study design

The framework was composed of three primary experiments (Fig. 2) designed to evaluate the performance and generalizability of pathological image-based models for HRD prediction. The experimental framework was outlined as follows.

#### Baseline HRD prediction for each candidate cancer type

Consistent with methodological precedents in computational histopathology^29^, we established a standardized validation framework (Fig. 2A) for histopathology-based HRD prediction by applying uniform cancer-specific training protocols across 10 candidate cancer cohorts (8 TCGA, 2 CPTAC). For each cancer type, the Baseline model was constructed as follows: UNI (ViT-L/16), pre-trained via DINOv2 self-supervised learning on 100 million pathology image tiles (Mass-100 K), served as the feature extractor; the CLAM-SB aggregation network was randomly initialized and trained from scratch on the respective target cancer dataset. Following the dataset splitting protocol described in Section “WSI preprocessing and dataset splitting”, cancer-specific CLAM-SB models were trained on the training subset, hyperparameter-tuned via the validation subset, and evaluated on the isolated test subset, generating five independent AUC scores for each cancer type. Mean and 95% confidence interval (CI) of AUC across the five splits were calculated to counteract stratification biases.

#### Selection of pretrained models

To determine the optimal pretrained model for transfer learning in this study, three key dimensions were systematically evaluated. First, clinical relevance was prioritized by focusing on cancer types with approved PARP inhibitors (PARPi), specifically ovarian cancer (OV), breast cancer (BRCA), prostate cancer (PRAD), and pancreatic cancer (PAAD). Second, data robustness criteria were applied, requiring a patient-level threshold (*n* ≥ 500) and an HRD-H positivity rate > 10%. This identified four candidate cancers: BRCA (*n* = 1,050), OV (*n* = 561) and LUAD (*n* = 507). Only BRCA and OV satisfied both clinical relevance and data volume thresholds, with ovarian cancer demonstrating the most established PARPi clinical adoption (FDA-approved since 2014) while breast cancer offered the largest sample size advantage. Third, comparative performance analysis evaluated OV and BRCA models using cross-cancer predictive efficacy, measured by AUC metrics, to finalize the optimal transfer learning pretrained model (Fig. 3B).

#### Comparison of HRD prediction with and without transfer learning

To evaluate whether HRD prediction for other cancers could benefit from the TCGA-BRCA model, we first fine-tuned it on each of the 7 candidate cohorts: TCGA cohorts (*n* = 7 cancer types, including LUAD, and SARC) and CPTAC cohorts (*n* = 2: LUSC, LUAD). We then compared model performance with and without transfer learning from the TCGA-BRCA model (Fig. 3C).

#### Model training and hyperparameters

The CLAM-SB model utilizes a gated attention network with a hidden dimension of 256 to aggregate 1024-dimensional instance features, regularized by a dropout rate of 0.25. For transfer learning, the model was fine-tuned from BRCA-pretrained weights with the classification head re-initialized. Training employed the Adam optimizer with a fixed learning rate of 2 × 10^-4^, weight decay of 1 × 10^-5^. The training strategy incorporated 5-fold cross-validation with early stopping within a maximum of 200 epochs. Loss functions comprised cross-entropy for slide-level classification and SVM for instance-level learning, with weighted sampling applied for class balancing.

### Model validation: molecular analysis and model interpretation

To comprehensively evaluate the model’s predictive capability for HRD status, our analysis extended beyond performance metrics to include molecular correlates and model interpretability.

#### Molecular analysis

To molecularly characterize the stratification results, we analyzed gene mutations and BRCA1 promoter methylation—two key drivers of HRD—using data from the TCGA-BRCA cohort. Gene mutation data (MC3 gene-level non-silent mutation) and DNA methylation data were obtained through the University of California Santa Cruz (UCSC) Genome Browser Database^58^ (http://genome.ucsc.edu). First, a mutation frequency analysis was conducted to assess the molecular correlates of the predictions. For this analysis, samples were stratified into HRD-H and HRD-L subgroups based on both the ground-truth labels and the model predictions. The alteration frequencies of key genes were then calculated and compared between these subgroups. In parallel, we evaluated the association between BRCA1 promoter methylation status and HRD status using a Chi-square test. Samples were dichotomized into methylation (β ≥ 0.07) and unmethylation (β < 0.07) groups based on methylation beta values from the 450 K array^59^. CpG probe identifiers (e.g., cg09441966) denote specific probes within the BRCA1 promoter region, with beta values indicating methylation intensity (continuous variables between 0 and 1, representing the ratio of methylated bead type intensity to combined locus intensity). These groups were cross-tabulated with the model-predicted HRD-H/HRD-L classifications in a 2 × 2 contingency table. The Chi-square test was then applied to determine the statistical significance of the association between methylation status and predicted HRD status.

#### Model interpretation

To interpret the model’s predictions using TCGA-BRCA, we performed a multi-level analysis of morphological features associated with HRD status, beginning at the whole-slide level and progressing to cellular-level examination.

At the whole-slide level, we employed the CLAM-SB framework^56^ to generate attention-based visualizations. Using the model’s built-in attention mechanism, we compute attention weights for individual tissue tiles within WSIs, quantifying each tile’s contribution to the HRD status prediction. These weights were visualized as heatmap overlays using a red-to-blue gradient, where red indicates high-attention regions supporting the predicted class. This explainable artificial intelligence (XAI) approach translates the model’s decision-making process into an intuitive format, enabling correlation between high-attention regions and biologically relevant morphological features.

Following the whole-slide analysis, we further performed cellular-level quantification and compared the abundance of various cell types between HRD-H and HRD-L tumors predicted by the TCGA-BRCA model. We selected the top 20 WSIs with the highest prediction scores for each group. For each WSIs, the 20 tissue tiles corresponding to the highest attention scores, as determined by the model, were extracted. These tiles were subsequently processed using CellViT++ (https://github.com/TIO-IKIM/CellViT-plus-plus) for cell segmentation and classification^46^. CellViT + + utilizes a ViT-Huge encoder pre-trained via the Segment Anything Model (SAM), followed by fine-tuning on the PanNuke dataset comprising nearly 200,000 labeled nuclei across 19 tissue types and 5 nuclei classes (Neoplastic, Epithelial, Inflammatory, Connective, and Dead cells). On this benchmark dataset, CellViT-SAM-H achieved per-class F1-scores of 0.71 (Neoplastic), 0.73 (Epithelial), 0.58 (Inflammatory), 0.53 (Connective), and 0.36 (Dead), supporting the reliability of our interpretability analysis. No additional domain adaptation or cancer-type-specific fine-tuning was performed; the model was applied off-the-shelf to ensure unbiased quantification of cellular composition. The five cell categories were defined as: “Connective”, “Dead”, “Epithelial”, “Neoplastic”, and “Inflammatory” cells. Finally, the Mann-Whitney U test was applied to compare the per-tile cell type counts between HRD-H and HRD-L groups, with a significance level of α = 0.05.

### Statistical analysis

Following previous studies on binary classification^29^, our study utilized the AUC for model performance evaluation (including baseline model performance, pretrained model, and transfer learning model outcomes). We adopted AUC, reported with a 95% CI, and the area under the precision-recall curve (AUPRC) as the primary statistical metrics of our study. To further evaluate model performance, an independent-samples t-test was employed to compare the patient-level predicted probabilities between the HRD-H and HRD-L groups, using a significance level of α = 0.05.

The model was trained for 200 epochs on a NVIDIA GeForce RTX 4090. All statistical analyses and machine learning evaluations were performed in Python version 3.10.12, utilizing the following libraries: scipy (version 1.14.1) for statistical tests, scikit-learn (version 1.5.2) for metric calculation (AUC, AUPRC), and pytorch (version 2.5.1 + cu124) for model training and inference.

## Supplementary Information

Below is the link to the electronic supplementary material.

Supplementary Material 1

Supplementary Material 2

Supplementary Material 3

Supplementary Material 4

Supplementary Material 5

Supplementary Material 6

Supplementary Material 7

Supplementary Material 8

## Data Availability

The data presented in this study are available in The Cancer Genome Atlas (TCGA) and the Clinical Proteomic Tumor Analysis Consortium (CPTAC) repositories. These data were derived from the following resources available in the public domain: https://portal.gdc.cancer.gov/ and https://www.cbioportal.org/. All the code is available at the github repository: https://github.com/wangzhengxiao/HRD-HE-Prediction.

## Abbreviations

AI: Artificial intelligence
AUPRC: Area under the precision-recall curve
AUROC: Area under the receiver operating characteristic curve
BER: Base excision repair
BLC: Basal-like breast cancer
BLCA: Bladder urothelial carcinoma
BRCA: Breast invasive carcinoma
BRCA1/2: Breast cancer genes 1 and 2
CI: Confidence interval
CNV: Copy number variation
CpG: Cytosine-phosphate-guanine
CPTAC: Clinical proteomic tumor analysis consortium
DL: Deep learning
DSB: DNA double-strand breaks
DDR: DNA damage repair
FDA: U.S. Food and Drug Administration
GATK: The Genome Analysis Toolkit
GIS: Genomic instability score
H&E: Hematoxylin and eosin
HNSC: Head and neck squamous cell carcinoma
HR: Homologous recombination
HRD: Homologous recombination deficiency
HRD-H: HRD high
HRD-L: HRD low
HRR: Homologous recombination repair
LOH: Loss of heterozygosity
IQR: Interquartile range
LST: Large-scale state transition
LUAD: Lung adenocarcinoma
LUSC: Lung squamous cell carcinoma
mCRPC: Metastatic castration-resistant prostate cancer
MIL: Multiple instance learning
MMR: Mismatch repair
MPP: Micro per pixel
NER: Nucleotide excision repair
NHEJ: Nonhomologous end-jointing
NtAI: Number of telomeric allelic imbalance
OV: Ovarian serous cystadenocarcinoma
PARP: Poly (ADP-Ribose)-polymerase
PARPi: Poly (ADP-Ribose)-polymerase inhibitor
PRC: Precision-recall curve
RCC: Renal cell carcinoma
SARC: Sarcoma
SBS3: Single base substitution 3
SNP: Single nucleotide polymorphism
STAD: Stomach adenocarcinoma
TAI: Telomeric allelic imbalance
TCGA: The Cancer Genome Atlas
WSI: Whole slide image
WSIs: Whole slide images
