# Supplementary material for: Cross-cancer homologous recombination deficiency prediction from whole slide images using transfer learning
Source: Sci Rep. 2026 May 12;16:21704. doi: 10.1038/s41598-026-52094-6 (PMC13358160; doi:10.1038/s41598-026-52094-6)
Supplement: Supplementary file 1 — Supplementary Material 1 [file 41598_2026_52094_MOESM1_ESM.docx]

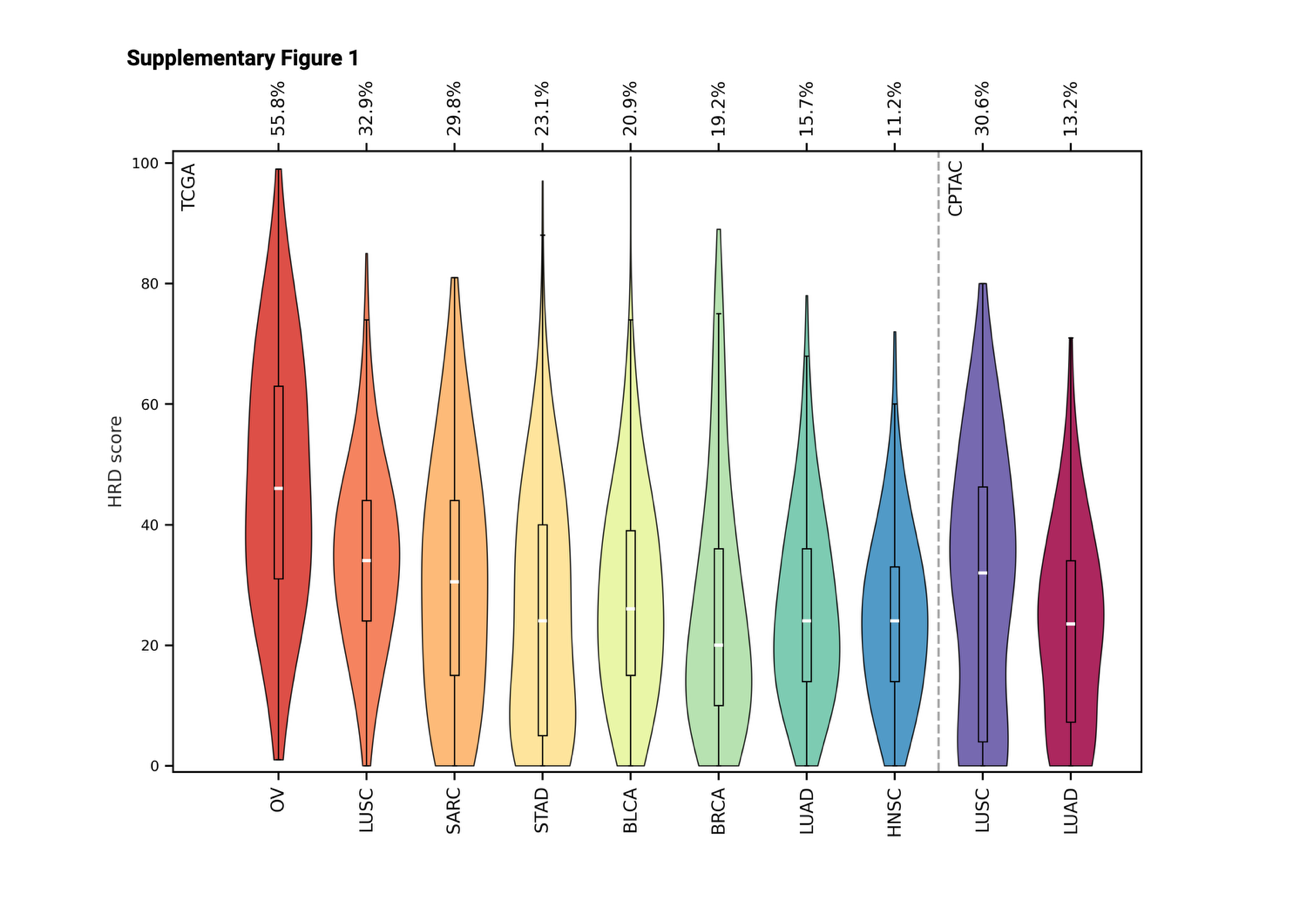


**Supplementary Figure 1** shows violin plots of HRD score distributions for each cancer type, with the percentage of HRD-high cases (using the common cutoff value of HRD score ≥ 42) indicated above each plot. The distributions reveal substantial heterogeneity in HRD prevalence across cancer types, ranging from 11.2% in HNSC to 55.8% in OV.


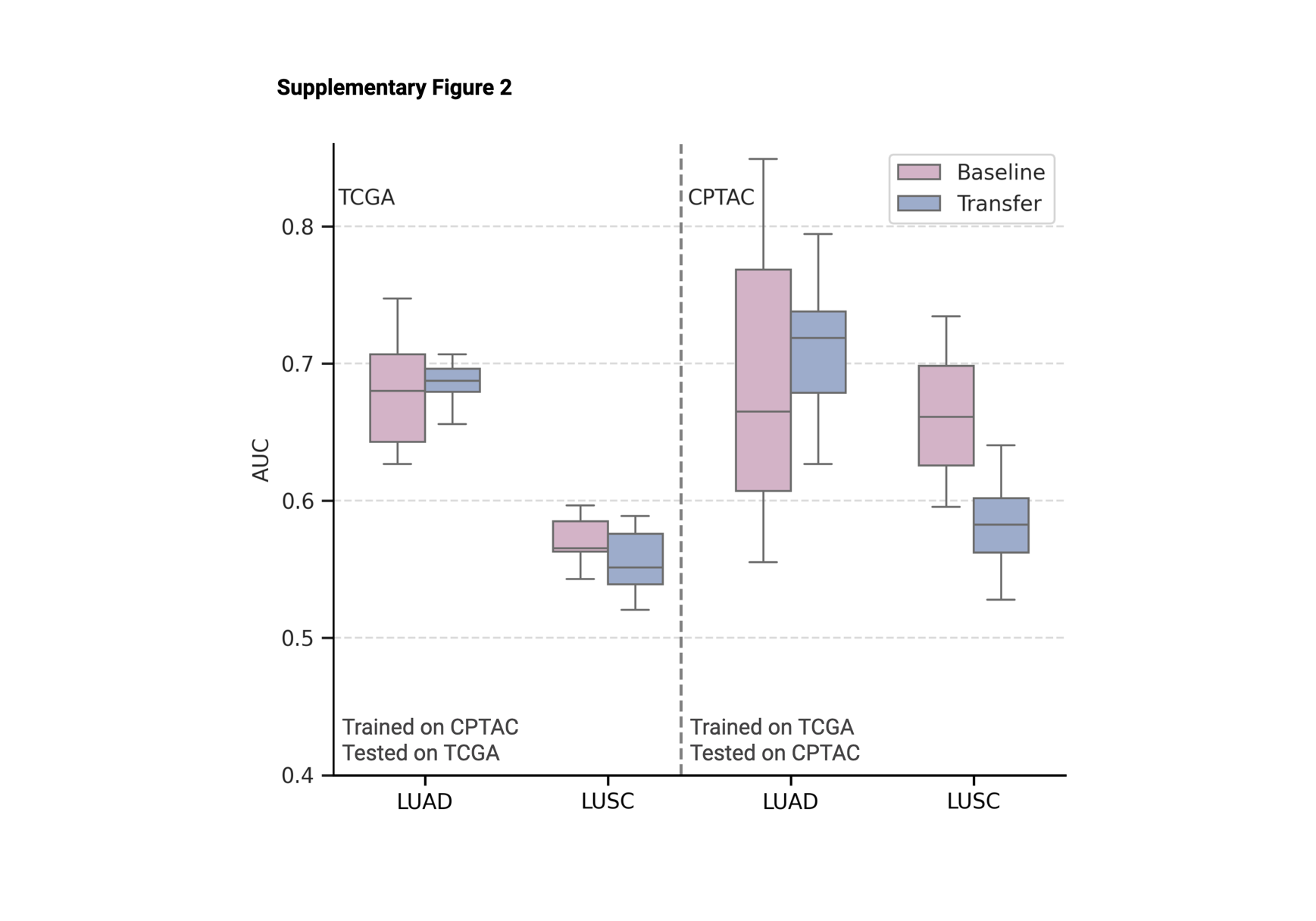


**Supplementary Figure 2:** **Cross-dataset validation of transfer learning for HRD prediction in lung cancer**. Box plots comparing prediction performance (AUC) between baseline models and transfer learning models across four cross-dataset validation scenarios. The left panel (CPTAC→TCGA) shows models applied to TCGA-LUAD and TCGA-LUSC test sets, where baseline models were trained from scratch on the respective TCGA dataset, whereas transfer learning models were initialized from weights pretrained on TCGA-BRCA and subsequently fine-tuned using the target TCGA dataset. The right panel (TCGA→CPTAC) shows models applied to CPTAC-LUAD and CPTAC-LUSC test sets, where baseline models were trained solely on the respective CPTAC dataset, whereas transfer learning models were initialized from the same TCGA-BRCA pretrained weights and fine-tuned using the target CPTAC dataset.


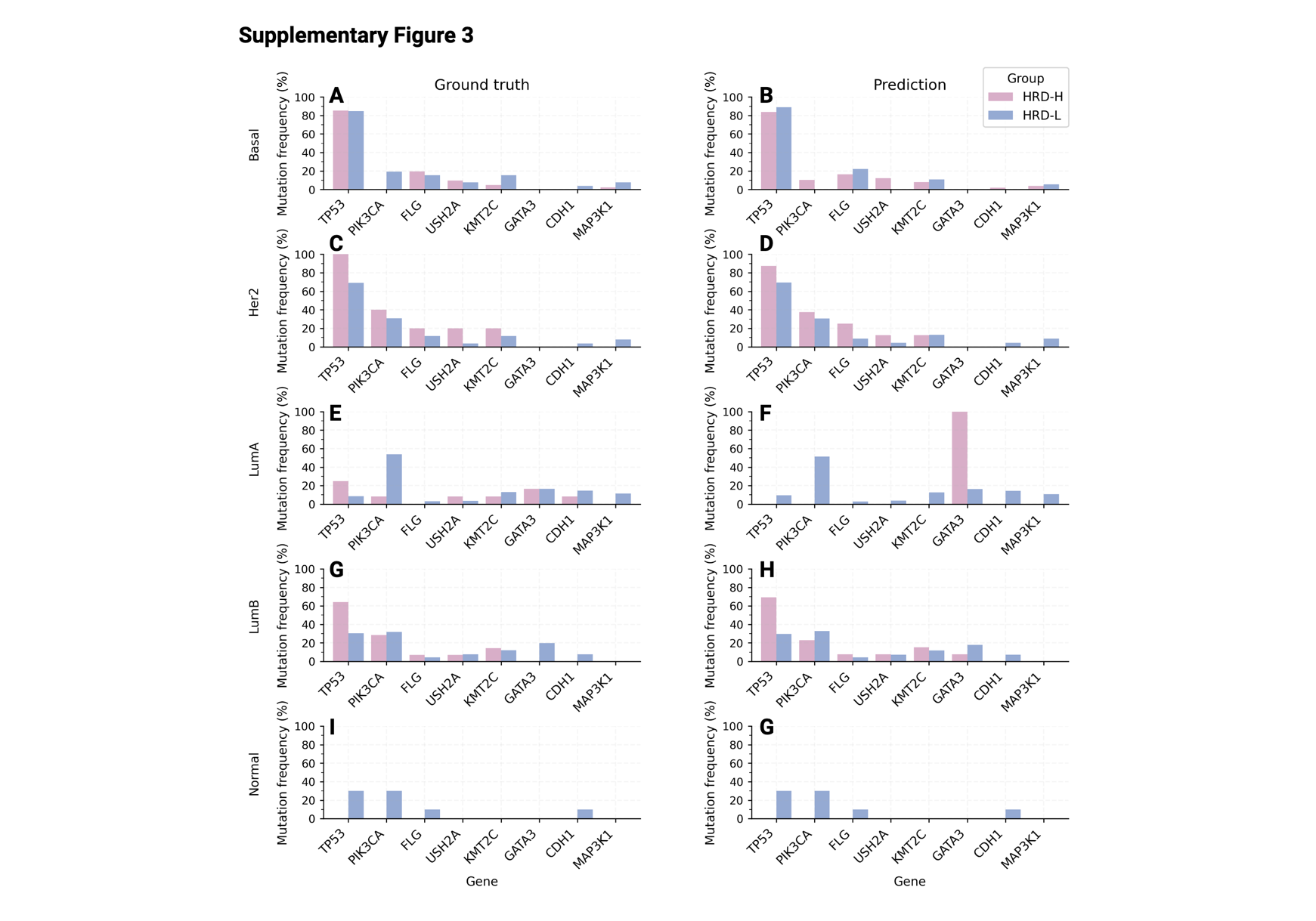


**Supplementary Figure 3** presents detailed mutational profiles stratified by PAM50 subtype. For each subtype (Basal, Her2, LumA, LumB, Normal), we compared mutation frequencies of key genes (TP53, PIK3CA, FLG, USH2A, KMT2C, GATA3, CDH1, MAP3K1) between HRD-H and HRD-L tumors, shown separately for ground truth labels (left panel) and model predictions (right panel). Within each subtype category, HRD-H tumors consistently show distinct mutational patterns compared to HRD-L tumors. For example, in the LumA subtype—where PIK3CA mutations are generally prevalent—HRD-L tumors exhibit markedly higher PIK3CA mutation rates than HRD-H tumors (53.77% vs. 8.33% in ground truth). Conversely, TP53 mutations are elevated in HRD-H tumors across multiple subtypes including Her2 and LumB. These within-subtype differences demonstrate that the model captures HRD-associated genomic features that are independent of the molecular subtype itself.


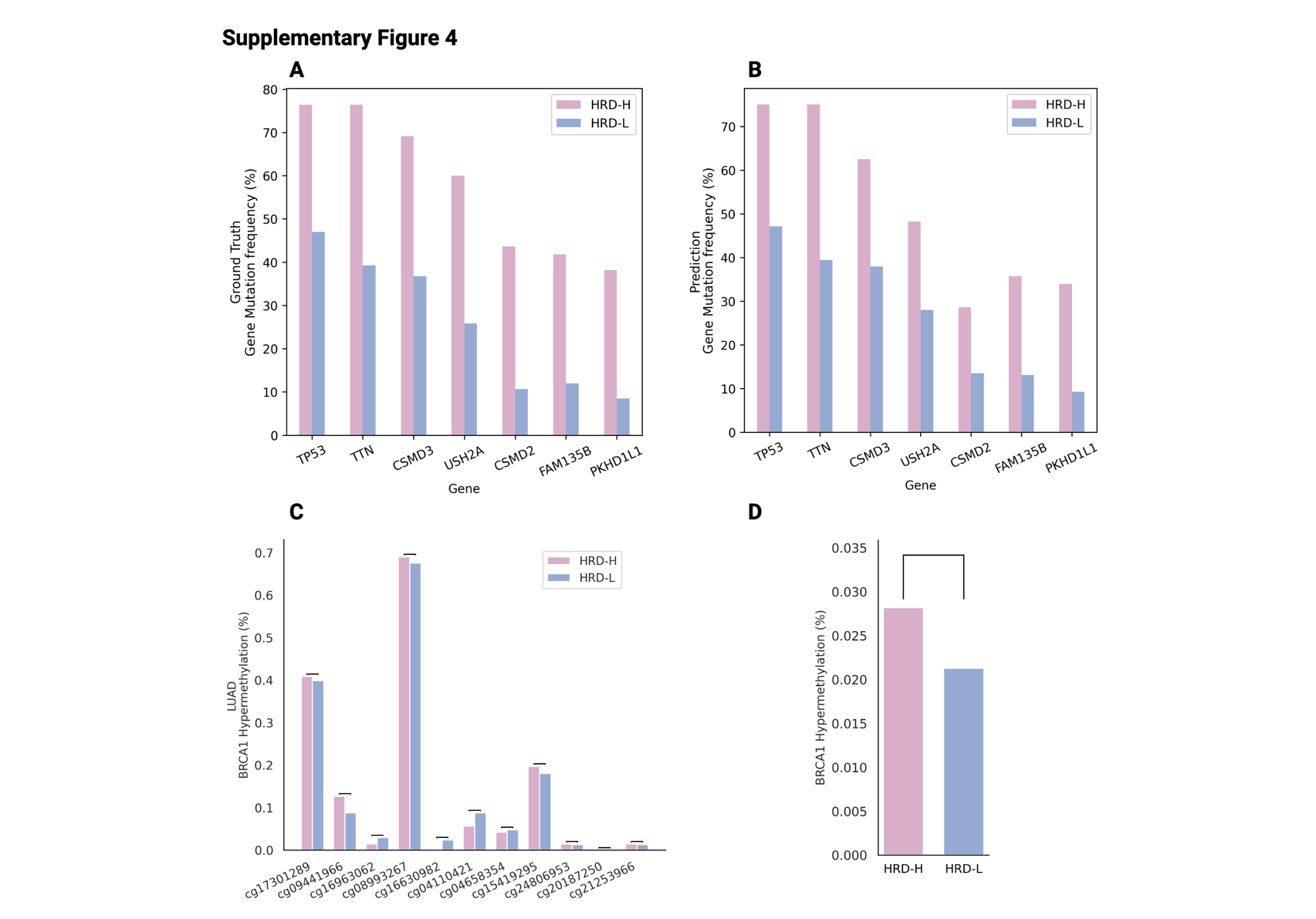


**Supplementary Figure 4**: Molecular Characterization of The Cancer Genome Atlas lung adenocarcinoma (TCGA-LUAD) cohort using the transfer learning model. Ground truth HRD labels were determined using a threshold of HRD score ≥ 42 for HRD-H and HRD score < 42 for HRD-L. (A, B) Gene mutation frequencies stratified by ground truth (A) and predicted (B) HRD status. (C, D) BRCA1 promoter methylation patterns: (C) probe-level methylation for ground truth HRD status; (D) gene-level comparison between HRD-H and HRD-L groups for predicted HRD stratification. Methylation levels of individual CpG probes within the BRCA1 promoter region are shown in (C), with beta values indicating methylation intensity. P-values calculated by chi-square test (D) or Wilcoxon rank-sum test (C); **p* < 0.05, ***p* < 0.01, ****p* < 0.001.
