# Supplementary material for: Cross-cancer homologous recombination deficiency prediction from whole slide images using transfer learning
Source: Sci Rep. 2026 May 12;16:21704. doi: 10.1038/s41598-026-52094-6 (PMC13358160; doi:10.1038/s41598-026-52094-6)
Supplement: Supplementary file 2 — Supplementary Material 2 [file 41598_2026_52094_MOESM2_ESM.docx]

**Supplementary Table 1**: The rates of Homologous Recombination Deficiency (HRD) positivity across 33 cancer types in The Cancer Genome Atlas (TCGA) cohort, showing the proportion of HRD-high (HRD-H) cases for each malignancy.

**Supplementary Table 2**: Data sources and sample characteristics from TCGA (BLCA, BRCA, SARC, etc.) and Clinical Proteomic Tumor Analysis Consortium (CPTAC) (LUSC, LUAD), presenting both patient-level and slide-level distributions of HRD-high (HRD-H), HRD-low (HRD-L), and total cases across cancer types.

**Supplementary Data 1**: Quantitative analysis of cellular features comparing HRD-H and HRD-L groups, based on the top 20 WSIs with the highest prediction scores from each group. **Sheet 1**: Detailed cell counts for the 20 highest-attention tiles from each of the 20 WSIs per group. **Sheet 2**: Summary statistics and comparative analysis of cellular features between HRD-H and HRD-L groups.

**Supplementary Data 2**: Patient-level HRD scores with corresponding Telomeric Allelic Imbalance (TAI), Loss of Heterozygosity (LOH), and Large-Scale Transitions (LST) values for specimens in TCGA (**Sheet 1**) and CPTAC (**Sheet 2**) datasets.

**Supplementary Data 3**: Performance comparison of the area under the receiver operating characteristic curve (AUROC, with 95% confidence interval), area under the precision-recall curve (AUPRC), Precision, Recall, and F1-score for baseline, zero-shot cross-cancer (TCGA-BRCA and TCGA-OV), and transfer learning models across eight cancer types (**Sheet 1**), and bidirectional cross-dataset validation between TCGA and CPTAC lung cancer cohorts (**Sheet 2**).

**Supplementary Data 4**: Provides the results for our head-to-head comparison. **Sheet 1**: Presents the per-cancer re-implementation results of the prior independent-training approach on our cohorts. **Sheet 2**: Summarizes the head-to-head comparison between prior study ( independent training，Ref. 29) and ours (independent baseline and BRCA-to-target transfer learning), stratified by transfer responsiveness. **Sheet 3**: Summarizes the head-to-head comparison of mean AUCs across cancer datasets, stratified by transfer responsiveness, between the prior study (independent training, Ref. 29) and ours (independent baseline and BRCA-to-target transfer learning).
